# True T1 mapping with SMART_1_Map (saturation method using adaptive recovery times for cardiac T_1_ mapping): a comparison with MOLLI

**DOI:** 10.1186/1532-429X-15-S1-P3

**Published:** 2013-01-30

**Authors:** Glenn S Slavin, Jeff A Stainsby

**Affiliations:** 1 GE Healthcare, Bethesda, MD, USA; 2 GE Healthcare, Toronto, ON, Canada

## Background

SMART_1_Map is a new single-point technique for cardiac T1 mapping [1]. Unlike Look-Locker approaches, such as MOLLI, which yield an "apparent" T1 (T1*), SMART_1_Map directly measures true T1. Because T1* is a function of imaging parameters, it is always shorter than T1, and correction methods are required to obtain the true T1. This work compared the accuracy of SMART_1_Map with MOLLI in phantom experiments under several imaging conditions.

## Methods

Analogous to the gold-standard spin-echo methods for T1 measurement, SMART_1_Map uses a series of single-point saturation-recovery experiments, each consisting of a saturation pulse, a delay time TS during which free T1 relaxation occurs, and a balanced SSFP readout. Short TSs (< RR interval) were acquired within a single heartbeat and were automatically and evenly distributed between TS_min_ and trigger delay. Longer TSs (free relaxation up to 4xRR), which were unachievable until now, were performed across multiple heartbeats. Although MOLLI assumes a constant heart rate for TIs that span several heartbeats, these long delay times are in fact heart-rate-dependent. In order to accurately quantify long TSs, SMART_1_Map adapts the actual recovery time to changing heart rates by measuring all heartbeats in real time. This makes SMART_1_Map insensitive to intra-scan heart rate variations. A phantom composed of a range of T1s and T1/T2 ratios was scanned with single-shot SMART_1_Map and MOLLI on a GE MR450w scanner. Five TSs were acquired in 13 heartbeats for SMART_1_Map and 8 TIs in 14 heartbeats for MOLLI (range: 100 to ~4000 ms) to determine T1* (MOLLI) and T1 (SMART_1_Map). T1* for MOLLI was corrected using a previously reported method [2]. Scans were repeated at simulated heart rates of 60, 75, and 100 bpm and with different readout window durations (T_acq_).

## Results

**Figure 1:**
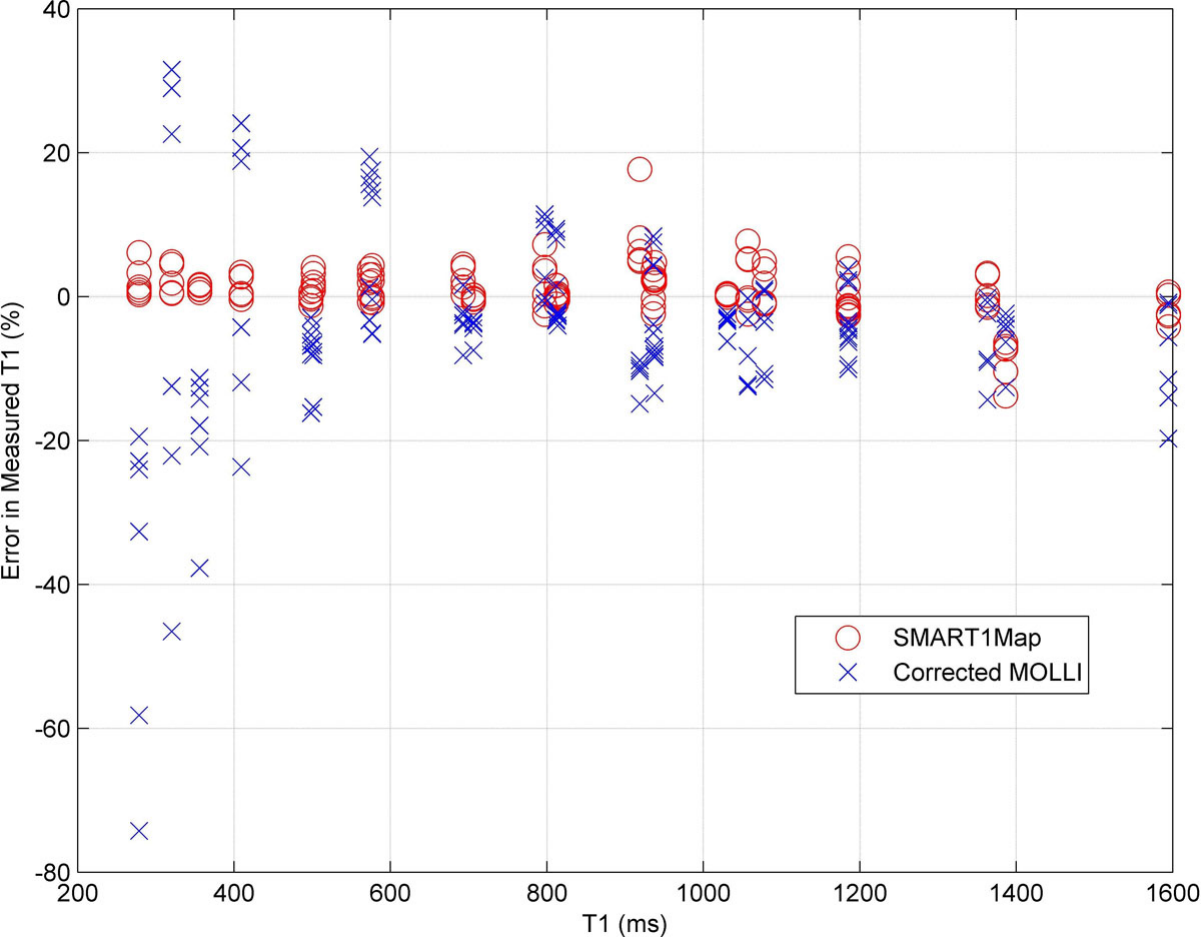
Percent error in measured T1 for all experiments from SMART_1_Map and MOLLI compared with reference T1 from IR spin-echo. The significantly higher errors for MOLLI at lower T1s could be problematic for post-contrast-enhanced imaging, where T1s are expected to be less than 500 ms.

**Figure 2:**
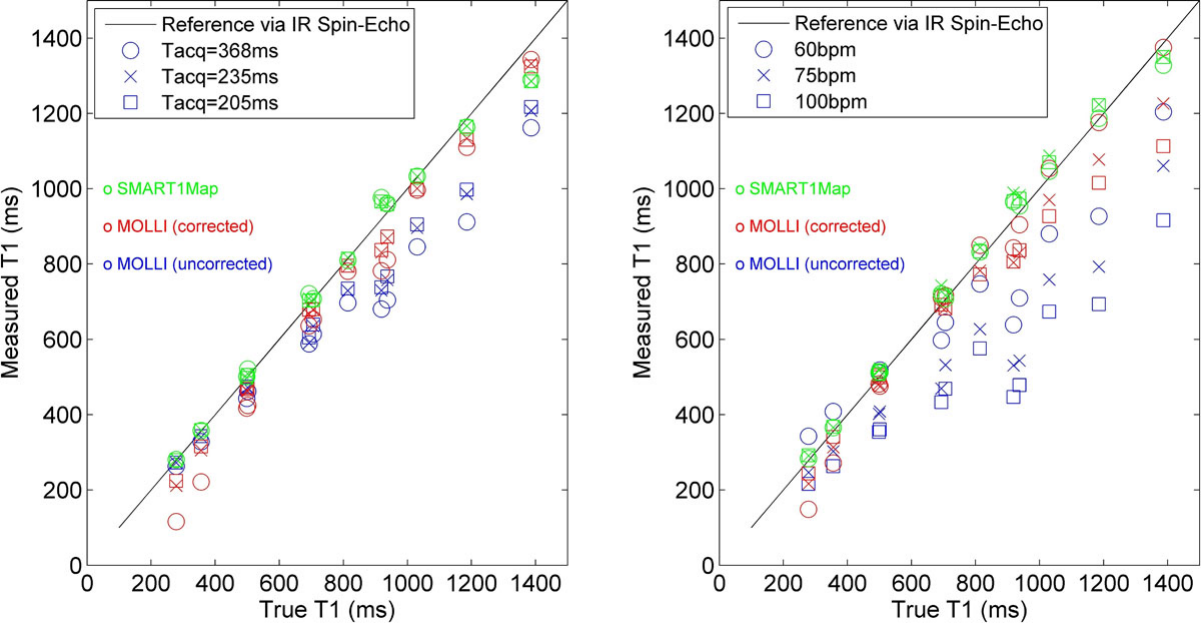
T1 accuracy for SMART_1_Map, uncorrected MOLLI, and corrected MOLLI as a function of readout window duration T_acq_ (left) and heart rate (right). In both cases, although corrected MOLLI (red) shows improvement over uncorrected MOLLI (blue), it still tends toward underestimation, especially at low T1s. In general, T1 underestimation with MOLLI increases with T_acq_, heart rate, and T1. In contrast, SMART_1_Map (green) exhibits consistent accuracy across the range of T1s and demonstrates no sensitivity to T_acq_ or heart rate.

Average T1 errors over all measurements were 0.9±3.4% for SMART_1_Map and -4.8±13.3% for MOLLI (Figure. 1). Comparisons of SMART_1_Map with MOLLI for variations in heart rate and T_acq_ are shown in Figure 2.

## Conclusions

SMART_1_Map yielded more accurate T1 measurements than MOLLI in all cases. As expected, T1* measured with MOLLI underestimated true T1. Although its applicability has yet to be validated, the MOLLI correction yielded improved results at some T1s but broke down at others. This is likely due to MOLLI's T2 dependence which is not accounted for in the correction. MOLLI also showed significant sensitivity to changes in heart rate and T_acq_. In contrast, SMART_1_Map demonstrated consistently accurate results that were independent of T1, heart rate, and T_acq_. Because SMART_1_Map is a single-point acquisition, it is insensitive to imaging parameters and requires no post-processing correction. In addition, the accuracy of the long delay times, which has previously been overlooked in cardiac imaging, is assured by measuring the duration of every heartbeat. As a result, SMART_1_Map should be robust to variable imaging conditions.

## Funding

N/A
